# Dynamics of extended-spectrum beta-lactamase-producing *Enterobacterales* colonization in long-term carriers following travel abroad

**DOI:** 10.1099/mgen.0.000576

**Published:** 2021-07-19

**Authors:** Laurence Armand-Lefèvre, Emilie Rondinaud, Dimitri Desvillechabrol, Jimmy Mullaert, Olivier Clermont, Marie Petitjean, Etienne Ruppe, Thomas Cokelaer, Christiane Bouchier, Olivier Tenaillon, Laurence Ma, Yasmine Nooroya, Sophie Matheron, The VOYAG-R Study Group, Antoine Andremont, Erick Denamur, Sean P. Kennedy

**Affiliations:** ^1^​ Laboratoire de Bactériologie, Hôpital Bichat-Claude Bernard, AP-HP Nord-Université de Paris, F-75018 Paris, France; ^2^​ Université de Paris, IAME, INSERM UMR 1137, F-75018 Paris, France; ^3^​ Plate-forme Technologique Biomics – Centre de Ressources et Recherches Technologiques (C2RT), Institut Pasteur, F-75015 Paris, France; ^4^​ Hub de Bioinformatique et Biostatistique – Département Biologie Computationnelle, Institut Pasteur, USR 3756 CNRS, F-75015 Paris, France; ^5^​ Service de Maladies Infectieuses et Tropicales, Hôpital Bichat-Claude Bernard, AP-HP Nord-Université de Paris, F-75018 Paris, France; ^6^​ Laboratoire de Génétique Moléculaire, Hôpital Bichat-Claude Bernard, AP-HP Nord-Université de Paris, F-75018 Paris, France; ^7^​ Département Biologie Computationnelle, Institut Pasteur, USR 3756 CNRS, F-75015 Paris, France

**Keywords:** *E. coli*, ESBL, carriage, long-term carriage, persistence, whole-genome sequencing, travel

## Abstract

Travel to tropical regions is associated with high risk of acquiring extended-spectrum beta-lactamase-producing *Enterobacterales* (ESBL-E) that are typically cleared in less than 3 months following return. The conditions leading to persistent carriage that exceeds 3 months in some travellers require investigation. Whole-genome sequencing (Illumina MiSeq) was performed on the 82 ESBL-E isolates detected upon return and 1, 2, 3, 6 and 12 months later from the stools of 11 long-term (>3 months) ESBL-E carriers following travel abroad. One to five different ESBL *Escherichia coli* strains were detected per traveller upon return, and this diminished to one after 3 months. Long-term carriage was due to the presence of the same ESBL *E. coli* strain, for more than 3 months, in 9 out of 11 travellers, belonging to epidemic sequence type complexes (STc 10, 14, 38, 69, 131 and 648). The mean carriage duration of strains belonging to phylogroups B2/D/F, associated with extra-intestinal virulence, was higher than that for commensal-associated A/B1/E phylogroups (3.5 vs 0.5 months, *P*=0.021). Genes encoding iron capture systems (*fyuA, irp*), toxins (*senB*, *sat*), adhesins (*flu, daaF, afa/nfaE*, *pap*, *ecpA*) and colicin (*cjrA*) were more often present in persistent strains than in transient ones. Single-nucleotide polymorphism (SNP) analysis in persistent strains showed a maximum divergence of eight SNPs over 12 months without signs of adaptation. Genomic plasticity was observed during the follow-up with the loss or gain of mobile genetic elements such as plasmids, integrons and/or transposons that may contain resistance genes at different points in the follow-up. Long-term colonization of ESBL-E following travel is primarily due to the acquisition of *E. coli* strains belonging to epidemic clones and harbouring ‘virulence genes’, allowing good adaptation to the intestinal microbiota.

## Data Summary

Raw sequence data and assemblies are available in the European Nucleotide Archive (EMBL-EMI) (http://www.ebi.ac.uk/ena) under project accession PRJEB41147 (sample accession numbers ERS5275910 to ERS5275994). The full list and characteristics of these strains are presented in Table S1 (available in the online version of this article) with the genome accession numbers.

**Impact Statement**

Travel to the tropics has long been associated with a high risk of intestinal acquisition of extended-spectrum beta-lactamase-producing *Enterobacterales* (ESBL-E). Because ESBL-E are typically eliminated rapidly upon return, long-term carriage remains an underexamined area. The present study explores the conditions giving rise to long-term ESBL-E carriage following travel as well as the underlying mechanisms. Our findings suggest that long-term carriage is primarily due to the carriage of a single persistent strain. This strain, we observe, is the result of neither the transfer of a plasmid-carrying ESBL to a commensal strain nor of a major adaptation to the host. Rather, we find that long-term carriage is a consequence of the acquisition of epidemic clones of *E. coli* mainly belonging to the phylogenetic groups B2/D/F and harbouring specific genetic traits (adhesins, iron capture systems, colicin and toxins) that make them immediately well adapted and allow them to successfully colonize their host.

## Introduction

The worldwide dissemination of multi-drug-resistant *Enterobacterales*, especially those producing extended-spectrum beta-lactamase (ESBL-E), is alarming. For nearly two decades, community carriage of ESBL-E has been steadily increasing [1]. However, this increase has been uneven over different regions of the world. The proportion of people carrying ESBL-E in their gut is estimated at 70 % in Asia and 45 % in Africa, but only 10 % in Europe [1]. It is recognized that travel from low-prevalence to endemic areas increases the risk of acquiring ESBL-E, with acquisition rates of ESBL-E varying from 20–50 %, depending on the geographical areas visited [2]. However, ESBL-E appear to be cleared quickly, as the median duration of carriage upon return is approximately 1 to 3 months [2–4]. Nevertheless, 5–10 % of travellers who acquire an ESBL-E remain carriers 6 or 12 months after return [2, 3]. The analysis of such long-term carriage is of major interest, as it certainly contributes to the enhanced spread of ESBL-E in communities of low-prevalence countries [3]. Previous studies have shown that longer carriage of travel-acquired ESBL-E is associated with several factors, including microbiota composition, high intestinal concentration of ESBL-E, Asian destination, previous antibiotic therapy, presence of *bla*_CTX-M9_ and colonization by *Escherichia coli* [2, 3, 5]. Indeed, among *Enterobacterales*, *E. coli* is the species best adapted species to the human intestinal microbiota [6]. Several studies, focusing rather on susceptible strains, have identified genetic factors, such as haemolysin, iron sensors and adhesins/fimbriae associated with long-term *E. coli* carriage [7, 8]. Persistent strains are also more likely to belong to the extra-intestinal virulent B2 phylogenetic group [7, 9]. However, these factors were inconsistently found in the various studies and have not been explored in multi-drug-resistant strain carriage. In a previous study aiming to assess the acquisition rates of multi-drug-resistant *Enterobacterales* in 574 subjects travelling in tropical areas, we showed that 51 % of travellers had acquired at least 1 ESBL-E and that the median duration of carriage after return was 1 month. However, we found that 11 of these travellers were still carriers 6 months after return [2]. Here, we describe the dynamics of ESBL-E colonization in these long-term carriers and the bacterial genomic factors associated with persistence.

## Methods

### General presentation of the study

This study is an ancillary study of the VOYAG-R clinical trial (clinicaltrials.gov number NCT01526187), which aimed to determine the acquisition rate of multi-drug-resistant bacteria after a trip to the tropics and the duration of carriage after return [2]. In 2013, 574 carriage-free travellers provided faecal samples upon return (M0) and in the case of ESBL-E acquisition, 1 (M1), 2 (M2), 3 (M3), 6 (M6) and 12 (M12) months later. Stool samples were plated directly on ChromID ESBL agar (bioMérieux, Marcy-l’Etoile, France) and bi-plate ESBL agar (AES Chemunex, Ivry-sur-Seine, France). Concurrently, an enrichment step with a brain heart infusion (BHI) broth supplemented with 1.5 mg l^−1^ cefotaxime and incubated overnight was performed and plated on ChromID ESBL agar. All colonies with distinct aspects (size, colour, shape) on each medium and at each time point were identified by mass spectrometry (MALDI biotyper, Bruker-Daltonics, Bremen, Germany) and tested for antibiotic susceptibility (amoxicillin, co-amoxiclav, ticarcillin, cefotaxime, ceftazidime, cefepime, cefoxitin, ertapenem, imipenem, gentamicin, amikacin, nalidixic acid, ofloxacin, co-trimoxazole, tetracycline, fosfomycin) using the disc diffusion method (http://www.eucast.org). Isolates with distinct susceptibility patterns were stored in glycerol at −80 °C [2].

### Definitions

A long term-carrier was defined as a traveller carrying an ESBL-E for more than 3 months after return.

A strain was considered persistent if found in the same traveller for more than 3 months. Transient strains were those observed only at return and never afterwards (<1 month).

Except for strains isolated at M0, the duration of carriage of the strains detected at a single follow-up point could not be calculated and was assigned the status ‘not determined’ (nd).

### Strain selection and characterization

All the ESBL-E isolates identified in the long-term carriers at each time point were studied. DNA was extracted using the EZ1 DNA tissue kit (Qiagen, Courtaboeuf, France). The phylogenetic group of *E. coli* was determined as described previously [10], resulting in their classification into eight groups (A, B1, B2, C, D, E, F and *Escherichia* cryptic clade I).

#### Whole-genome sequencing (WGS) and subsequent analyses

DNA libraries were prepared using the Illumina Nextera Kit (Illumina, San Diego, USA). Pooled libraries were sequenced on an Illumina MiSeq instrument. *De novo* assemblies were created with a pipeline provided within the Sequana project [11], which uses SPAdes (v3.9.0) software [12]. The quality of the *de novo* assemblies was estimated using standard metrics computed using QUAST (v4.3) [13]. Reads used to construct the assemblies were remapped against the assembly contigs to visualize coverage and detect assembly errors.

Parsnp (v1.2) [14] and PhyML (v3.3.20180621) [15] software were used to construct a maximumlikelihood phylogenetic tree. The Center for Genomic Epidemiology website was consulted to identify sequence types (STs) and ST complexes (STc), serotypes, antimicrobial resistance genes, plasmid replicon and ST [plasmid multilocus sequence typing (pMLST)] (http://www.genomicepidemiology.org). Virulence factor genes were detected using Abricate software (v0.9.8) [16]. Localization of the ESBL-encoding genes was predicted by PlaScope (v1.3.1) [17]. Strains were thus characterized using the clone definition, based on the unique combination of phylogroup, Warwick University scheme ST, Pasteur Institute scheme ST, serotype, *fimH* allele and ESBL enzyme.

Within the same clone, the entire genomes of the isolates were compared. First, single-nucleotide polymorphisms (SNPs) were detected on the common genome of each clone using the variant calling pipeline from the Sequana project designed according to the best practices recommended by the Broad Institute [18]. Insertions/deletions were realigned using GATK3 (v3.8) Indel Realigner software, duplicated reads were ignored, and aligned reads with a mapping quality score <30 were removed (BWA score) [19]. Variant calling was performed with Freebayes (v1.0.2) using the default options except for the ploidy option, which was set at 1. Variants were filtered, and those with a Freebayes score >100, a frequency of 80%, a minimal depth of 10, and a minimal forward and reverse strand ratio of 0.2 were retained. Mutations were confirmed using the Integrative Genomics Viewer (IGV) tool (v2.3.8). Second, a global genome comparison was performed using a k-mer approach. This approach takes into account both SNPs and the presence/absence of genes. Genetic distances between all the genomes were calculated using ‘mash dist’ function in Mash software (v2.27.1) with a k-mer size of 32 and a sampling size of 5000 [20]. A tree based on the distance matrix was constructed.

Annotations of the genome were performed using RAST software [21] via the PATRIC Platform (v3.0) (https://www.patricbrc.org/) [22] and using the Microbial Genome Annotation and Analysis Platform (MaGe) (v3.5) [23]. Genes functions were checked on Uniprot (https://www.uniprot.org/).

#### Mutation analysis

To compute the ratio of synonymous to non-synonymous mutation rates, we counted the number of synonymous sites (S) and non-synonymous sites (N) in two diverged genomes of *E. coli*: strain 536 (B2 phylogroup) and REL606 (A phylogroup) [24]. We computed the rates as the total number of mutations observed (s for the synonymous, n for the nonsynonymous) divided by the number of possible sites, and the ratio of rates was computed as $R=\frac{n/N}{s/S}$.

### Statistical analysis

Based on the carriage status at each time point, the carriage duration was estimated as the number of months during which the strain was detected. Strains isolated on return (M0) were assigned a carriage duration of 0.5 months. As an additional sensitivity analysis, we also compared (alternative method) the carriage durations, considering that all strains isolated up to M3 were present at M0 (i.e. acquired during the journey).

Carriage durations were compared between strains belonging to the commensalism-associated A/B1/E phylogroups and the extra-intestinal virulence-associated B2/D/F phylogroups using the non-parametric Wilcoxon rank sum test. The rationale of this grouping is based on epidemiological and animal model experimental data [6, 25, 26].

The proportion of the presence/absence of virulence genes was compared between persistent and transient strains in a targeted approach. The *P*-value of the Fisher exact test was reported for all 149 genes of the panel. Due to the relatively low number of strains in each category, no multiple testing corrections were performed, and it was determined that the reported *P*-value should be used for gene ranking and not for the evaluation of statistical significance.

The same analysis was performed for the non-targeted approach, using the genes identified by the RAST and MaGe annotation tools. All genes that were not part of the core genome (genes of the variable genome) were included in the comparison analysis. In case of discrepancies, the presence of the gene was confirmed by blast on MaGe software.

## Results

Among the 292 travellers who acquired a multi-drug-resistant *Enterobacterales* during their trip, 11 (3.8 %) were long-term carriers (6 travellers were still carriers at M6 and 5 at M12). Eight individuals reported having travelled to Asian countries, while three travelled to Latin America. The median duration of travel was 18 days [interquartile range (IQR), 15.5–22 days]. Six of them reported taking antibiotics once or twice during the follow-up. (Table S1).

### Dynamic of colonization

During the follow-up period, 82 multi-drug-resistant *Enterobacterales* isolates, all ESBL *E. coli,* were isolated in the 11 long-term carriers (Table S1). The mean (range) number of isolates detected per traveller was 2 (1–5) at M0, 1.5 (1–3) at M1, 1.3 (1–2) at M2, 1.1 (1–2) at M3 and 1 at M6 and M12.

**Fig. 1.**
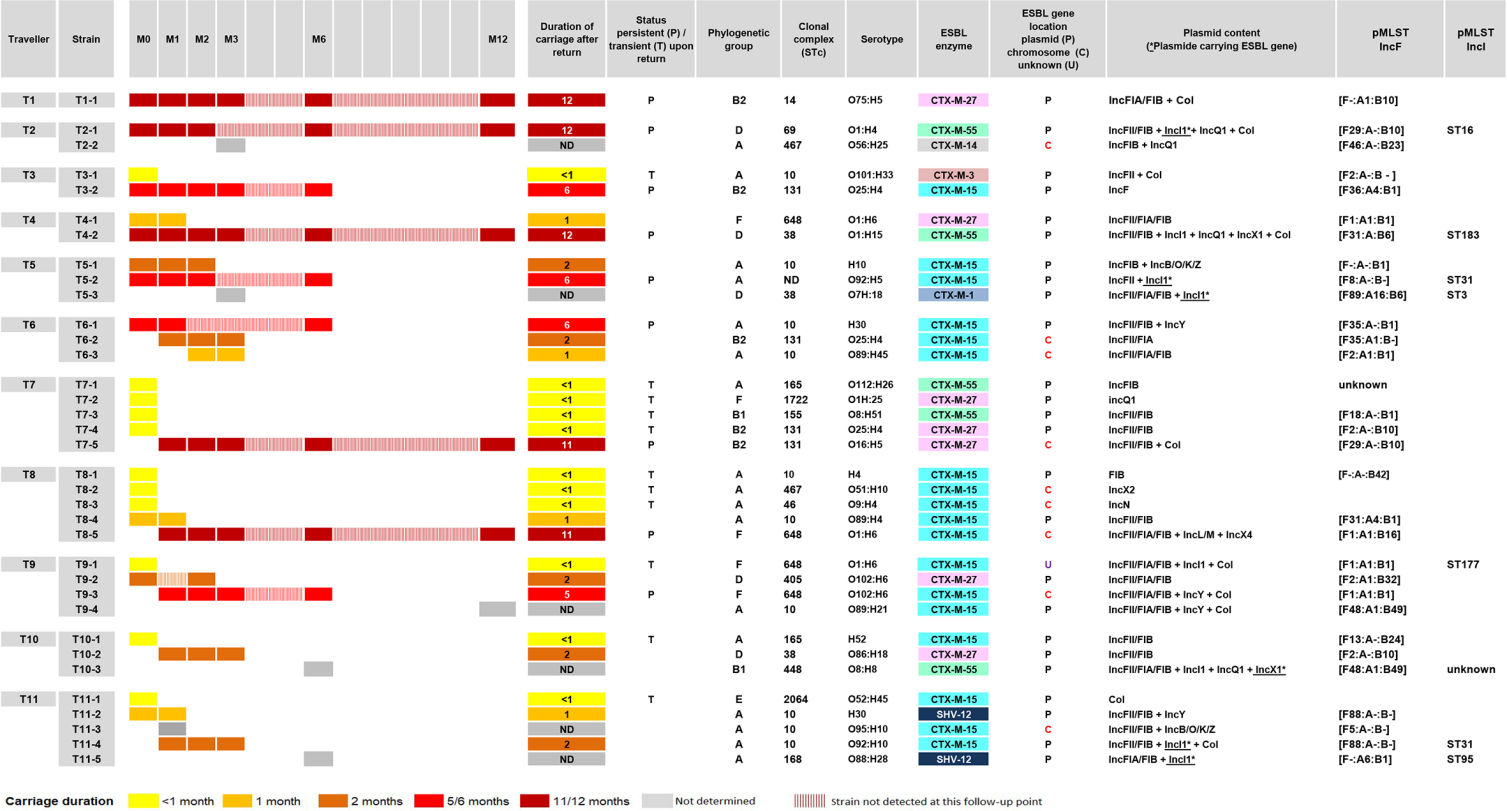
Follow-up of ESBL *E. coli* carriage in travellers after return.

The analysis of genomes of the 82 isolates identified 35 genetically different strains (clones) (Fig. 1, Table S1). The duration of carriage of the strains ranged from 0.5 to 12 months (median 1 month). Eleven strains were considered transient (duration of carriage: 0.5 months) and nine persistent (>3 months). The remaining had, for 10, an estimated duration of carriage between 1 and 3 months and for 5, a duration of carriage undetermined because isolated only at a single point of the follow-up (Fig. 1).

In 9 out of 11 travellers, long-term carriage was due to the presence of the same strain for more than 3 months (persistent strain). Indeed, in two travellers (T1 and T2), a single strain was isolated upon return and persisted for 12 months. In four (T3 to T6), several strains were isolated upon return but only one persisted. In three (T7 to T9), several strains were isolated upon return, but the persistent strain was isolated at M1. Last, in two travellers (T10 and T11) the long-term carriage was due to the acquisition at M6 of a new ESBL-E that had not previously been detected, and no persistent strains were observed.

### Global characteristics of strains

The 35 strains were distributed in 16 STc; the most frequent was STc10 (*n*=10), followed by STc131 and STc648 (*n*=4), STc38 (*n*=3), and STc 165 and 467 (*n*=2), with the others being singletons.

The genes encoding ESBL enzymes were predominantly *bla*_CTX-M_ genes (*n*=33, 94 %) and were mostly predicted to be plasmidic (*n*=25, 71 %). In all, 66 plasmids were detected in the 35 strains, and these were primarily IncF type (49 %, *n*=32), followed by IncI type (12 %, *n*=9) (Fig. 1).

**Fig. 2.**
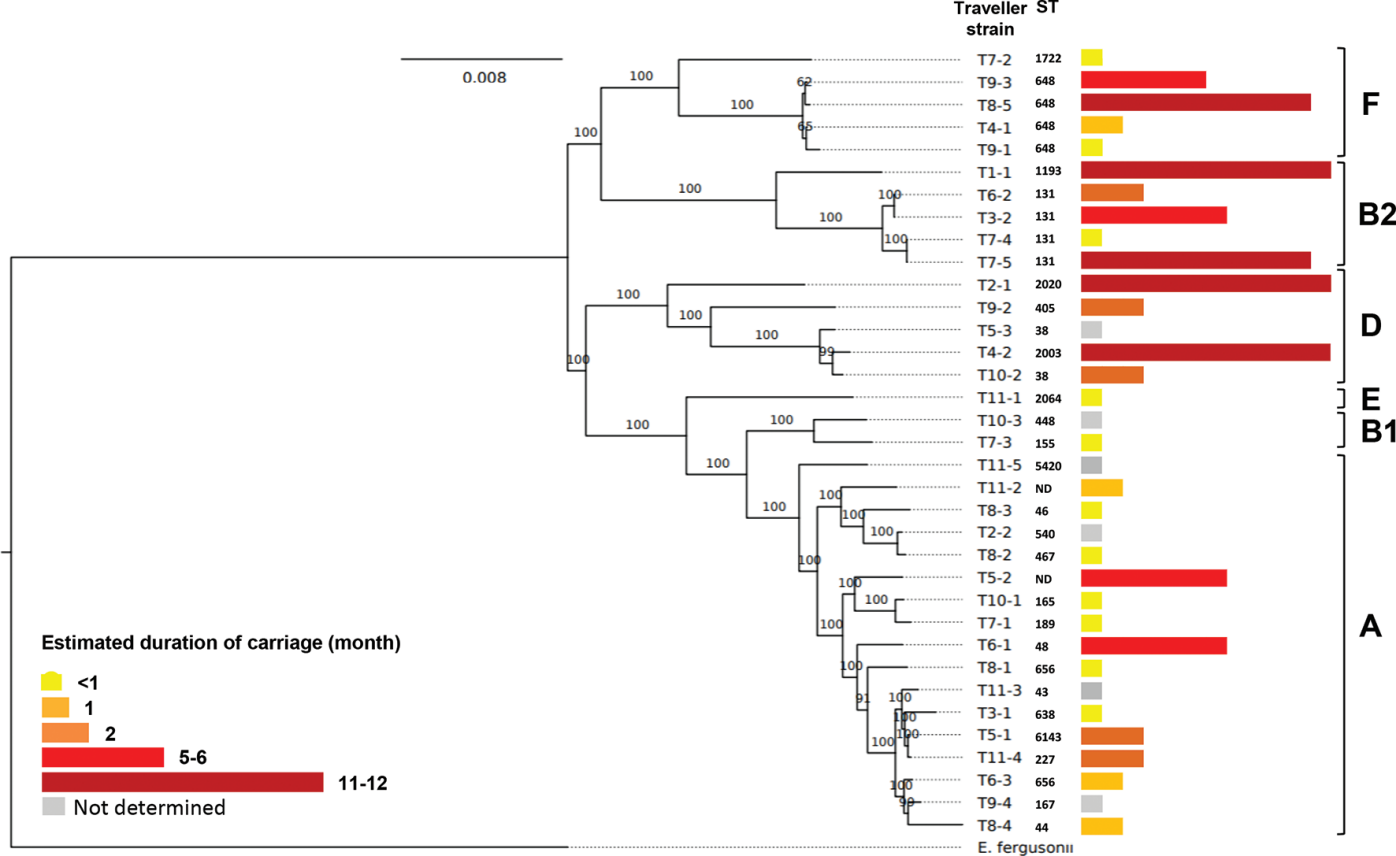
Estimated duration of carriage depending on the phylogenetic group of the ESBL *E. coli* strains. The phylogenetic tree was generated using the maximum-likelihood method. The tree was rooted on *Escherichia fergusonii*. The bootstrap values (1000 replicates) are shown near the nodes. Scale bar represents the number of nucleotide substitutions per site.

Fifteen (43 %) of these 35 different strains belonged to B2/D/F extra-intestinal virulent phylogroups, the median carriage duration of which was higher than that of those belonging to commensal A/B1/E phylogroups (3.5 vs 0.5 months, *P*=0.021). The significance of the difference remained when the alternative method for the estimation of carriage (i.e. from the return) duration was used (4.5 vs 0.5, *P*=0.018) (Fig. 2).

### Transient and persistent strain comparison

In order to investigate genetic traits potentially associated with persistence, the 9 persistent strains were compared to the 11 transient strains. Due to a risk of misclassification of the other strains resulting from intermittent carriage or lack of sensitivity of the culture, they were excluded from the comparison analysis.

#### Phylogenetic background

The persistent strains belonged primarily (*n*=7/9, 78%) to extra-intestinal virulence-associated phylogenetic groups B2 (*n*=3), D (*n*=2) and F (*n*=2), whereas the transient strains fell mostly (8/11, 72.7%) within commensal phylogenetic groups A (*n*=6), B1 (*n*=1) and E (*n*=1). Furthermore, the persistent strains mainly belonged to extra-intestinal pathogenic *E. coli* (ExPEC) epidemic clonal complexes, often reported as ESBL-E, including STc131 (*n*=2), STc648 (*n*=2), STc14/ST1193, STc69 [clonal group A (CGA)] and STc38. Transient strains were found distributed across 10 different STc (Fig. 1).

#### Targeted approach: plasmid, antibiotic resistance and virulence genes

No differences were found between the 12 types of plasmids isolated in the 9 persistent and the 11 transient strains. Of 42 different detected resistance genes, only the *qnrS* gene, coding for the quinolone resistance, was found more frequently in transient compared to persistent strains (Table 1).

**Table 1.** List of genes for which a different occurrence was found between persistent and transient *E. coli* strains by the targeted approach (among 47 resistance genes and 149 virulence genes) and the non-targeted approach (whole-genome comparison). Genes detected by both approaches are underlined and in bold. The reported *P*-value should be used for gene ranking and not for the evaluation of statistical significance

Genes	Function	Persistent strains, *n*=9 (%)	Transient strains, *n*=11 (%)	P*
**Resistance gene**				
***qnr***	Fluoroquinolone resistance	0	5 (45)	0.038
**Virulence genes**				
***fyuA*** *, irp1, **irp2**, irp3, irp4, irp5*	Yersiniabactin iron transporter gene cluster (HPI)	9 (100)	4 (36)	0.005
*ydeA, ydfA*	Active transporter	5 (56)	0	0.008
*shiA*	Shikimate transporter	8 (89)	3 (27)	0.010
*gfcA, gfcB, gfcC, gfcD, gfcE, etp, etk*	Capsule biosynthesis gene cluster	1 (11)	8 (73	0.010
*flu*	Antigen 43	9 (100)	5 (45)	0.014
*sgcA, sgcB, sgcC, sgcE, scgQ, sgcR, sgcX, yjhF, yjhG, yjhH, yjhL,yjhP, yjhU*	d-xylonate dehydratase gene cluster	6 (67)	1 (9)	0.017
***sat***	Serine protease autotransporter toxin	6 (67)	1 (9)	0.017
Unnamed	Fe2 +ABC transporter	7 (78)	2 (18)	0.022
*scsC, scsD*	Suppressor for copper tolerance or thioredoxin	7 (78)	2 (18)	0.022
***papG*** *, papF, papE, papK, papJ, papD, **papC**, papH papA, papI, papB*	*pap* gene cluster	4 (44)	0	0.026
*afaA, afaB, afaC, afaD, afaF /**nfaE***	Afimbrial adhesin	4 (44)	0	0.026
***daaF***	F1845 diffuse adherence adhesin DaaF	4 (44)	0	0.026
***senB***	Enterotoxin TieB protein	4 (44)	0	0.026
*cjrA, cjrB, crjC*	Colicin Ia	4 (44)	0	0.026
*impG/vasA, impH/vasB*	Type VI secretion system	8 (89)	4 (36)	0.028
***ecpR, ecpA, ecpB, ecpC, ecpD, ecpE***	*E. coli* common pilus operon	9 (100)	6 (55)	0.038
*mtnA, mtnK*	Methylthioribose phosphatase and isomerase	5 (56)	1 (9)	0.050

*The reported *P*-value should be used for gene ranking and not for the evaluation of statistical significance.

Of 149 genes known to be involved in virulence, 7 genes or operons/gene clusters were found more frequently in persistent strains than in transient ones. Four of them are involved in adhesion: the *papG* and *papC* genes belonging to the *pap* operon encoding P fimbriae, *nfaE* encoding a non-fimbrial adhesin, *daaF* encoding a diffuse adherence adhesin and the *ecpA,B,C,D,E* genes encoding *E. coli* common pilus. Two genes are involved in iron capture, the genes *fuyA* and *irp2* encoding the yersiniabactin siderophore and its transporter and belonging to the high pathogenicity island (HPI). The two remaining genes *sat* and *senB* encode toxins (Table 1).

#### Non-targeted approach

We also employed a non-targeted approach by searching for associations between any gene and the characteristics of the strains, persistent or transient. The genomes of 20 strains (9 persistent and 11 transient) were annotated. The RAST tool found a total of 6724 annotated genes, of which 3020 were part of the variable genome and were included in the comparison analysis; the MaGe tool found a total of 4535 annotated genes, of which 1835 (variable genome) were included. Hypothetical or poorly characterized genes, and genes from phage genomes, were then excluded from further analysis.

The genes that were found to have the greatest difference in the two types of strains are listed in Table 1. The untargeted method retrieved all of the genes previously identified with the targeted method (resistance and virulence genes). The *nfaE* gene was identified as *afaB* gene (97 % homology) encoding for an afimbrial adhesin. This method also identified additional genes that were more frequent in persistent strains, such as the *flu* gene encoding the antigen 43 involved in adhesion, type VI secretion system genes, an unnamed operon involved in iron transport, the *cjrA/cjrB* encoding a colicin and four genes encoding metabolic functions (Table 1).

### Evolution of persistent strains during follow-up

#### SNP-based comparison

**Fig. 3.**
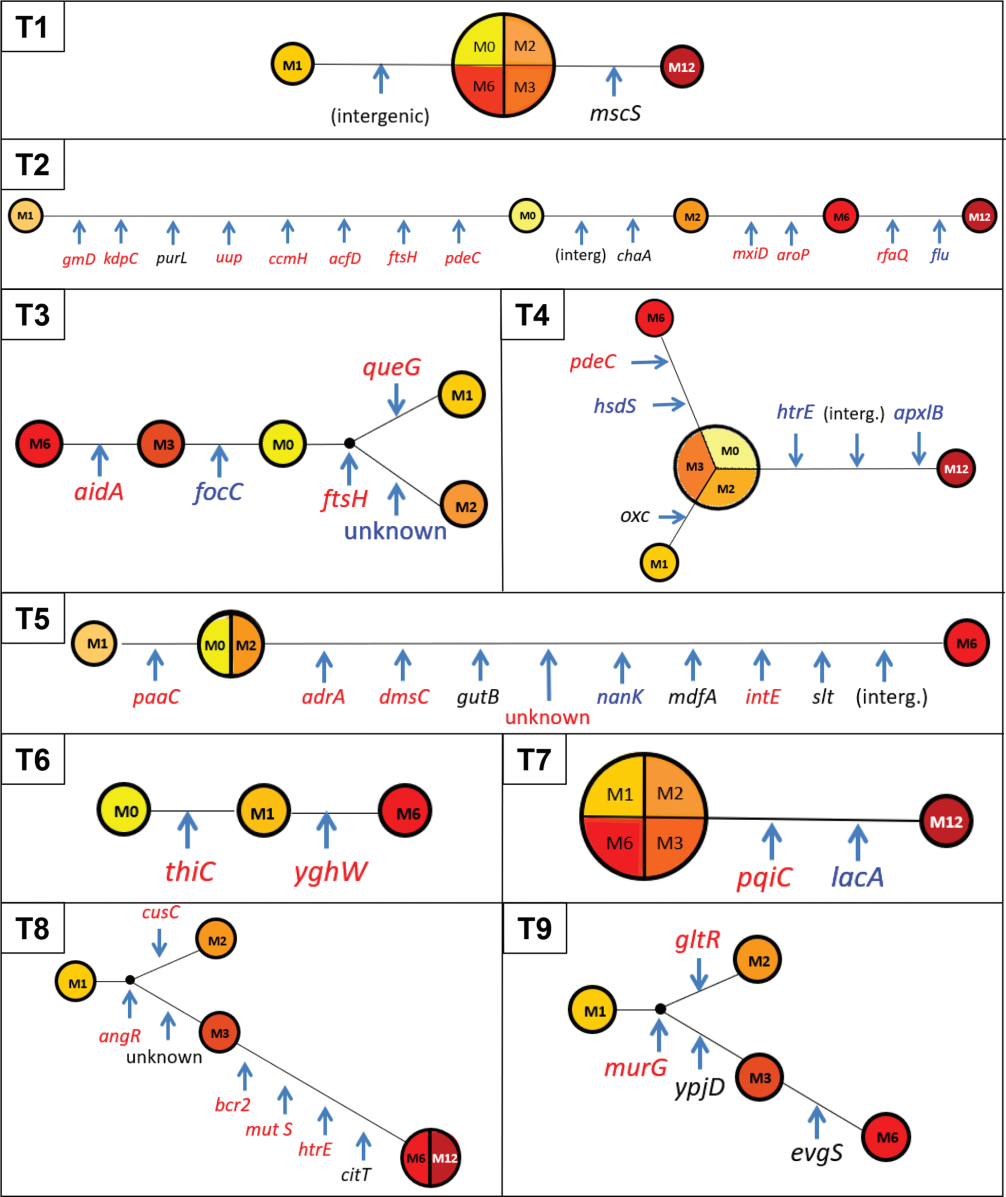
Mutations or frameshift events observed in the genome of persistent ESBL *E. coli* strains during follow-up. We created, for each traveller, a maximum-likelihood unrooted tree relating all isolates sampled during the follow-up period. For each traveller, the length of the branches is proportional to the number of mutations separating two samples. The diameters of circles are proportional to the numbers of isolates that are identical. Genes in red had non-synonymous mutation, genes in black had synonymous mutation and genes in blue had nonsense mutation or frameshift. This representation does not follow any time scale.

Compared to the first isolate (M0 or M1), from zero to a maximum of eight SNPs were observed in persistent strains at each time point of the follow-up. Of the 52 genetic events observed, 48 (92 %) were in coding regions (11 frameshifts and 37 mutations). These genes were affected with very few convergences, as only the *htrE* gene, encoding an outer-membrane usher protein, was mutated in two different persistent strains. Thirteen (27 %) genetic events were detected at more than one follow-up point and thus considered to be fixed. Among the 37 genetic mutations, 26 (70 %) were non-synonymous and 11 (30 %) synonymous (Table 2, Fig. 3). We computed a ratio of non-synonymous to synonymous rate of ~0.81 using all detected mutations. This value, close to 1, suggests that non-synonymous mutations accumulate at the same rate as synonymous mutations and are therefore not subject to active selection.

**Table 2.** Mutation and frameshift events observed in the genome of the persistent ESBL *E. coli* strains during the follow-up and their impact

Traveller	Gene	Synonym	Function of the encoded protein	Event	Mutation type	Fixed	M0	M1	M2	M3	M6	M12
**T1**	*mscS*		Mechanosensitive channel	C>T	Synonymous	No	Ref					X
	intergenic			Deletion AC	Intergenic	No	Ref	X				
**T2**	*mxiD*	*yscC,hrcC, invG*	Type III secretion outer-membrane pore-forming protein	C>G	Non-synonymous	Yes	Ref				X	X
	*pdeC*	*ylaB, yjcC*	Cyclic-di-GMP phosphodiesterase	A>T	Non-synonymous	No	Ref	X				
	*ftsH*		ATP-dependent zinc metalloprotease	T>A	Non-synonymous	No	Ref	X				
	intergenic			Insertion A	Intergenic	Yes	Ref		X		X	X
	*ccmH*	*yejP*	Cytochrome c-type biogenesis protein	T>A	Non-sense	No	Ref	X				
	*uup*	*ycbH, ycbI*	ABC transporter ATP-binding protein	T>A	Non-synonymous	No	Ref	X				
	*purL*		Phosphoribosylformylglycinamidine synthase	C>T	Synonymous	No	Ref	X				
	*kdpC*	*kac*	Potassium-transporting ATPase C chain	A>T	Non-synonymous	No	Ref	X				
	*aroP*	*ansP*	Aromatic amino acid transport protein	C>T	Non-synonymous	Yes	Ref				X	X
	*chaA*		Sodium–potassium/proton antiporter	T>A	Synonymous	Yes	Ref		X		X	X
	*acfD*	*yghJ*	Putative lipoprotein accessory colonization factor	T>A	Non-synonymous	No	Ref	X				
	*rfaQ*	*waaQ rfaQ, hepIII*	Lipopolysaccharide core heptosyltransferase	T>A	Non-synonymous	No	Ref					X
	*flu*	*yzzX, yeeQ, agn*	Antigen 43	Deletion TTC	Frameshift	No	Ref					X
	*gmd*	*yefN, yefA*	GDP-mannose 4,6-dehydratase	A>C	Non-synonymous	No	Ref	X				
**T3**	*focC*		Chaperone FimC	Insertion T	Frameshift	Yes	Ref			X	X	
	*aidA*	*yfaL*	Adhesine DNA-3-methyladenine glycosylase	C>T	Non-synonymous	No	Ref				X	
	*queG*		Epoxyqueuosine reductase	C>T	Non-sense	No	Ref	X				
	*ftsH*		Cell division protein	C>A	Non-synonymous	Yes	Ref	X	X			
	unknown		Hypothetical protein	Deletion G	Frameshift	No	Ref		X			
**T4**	*hsdS*	*hss*	Type I restriction–modification system, subunit S	Insertion AG	Frameshift	No	Ref				X	
	*htrE*	*yehB*	Outer-membrane usher protein	Insertion G	Frameshift	No	Ref					X
	*oxc*	*yfdU*	Oxalyl-CoA decarboxylase	C>T	Synonymous	No	Ref	X				
	intergenic			C>T	Intergenic	No	Ref					X
	pedC	*ycjG*	l-alanine-dl-glutamate epimerase	C>T	Non-synonymous	No	Ref				X	
	*apxIB*	*lapB*	Type I secretion system ATPase	Deletion G	Frameshift	No	Ref					X
**T5**	*adrA*	*dgcC, yaiC*	Diguanylate cyclase	T>A	Non-synonymous	No	Ref				X	
	*dmsC*	*yfnH*	Anaerobic dimethyl sulfoxide reductase, chain C	C>G	Non-synonymous	No	Ref				X	
	*paaC*		1,2-phenylacetyl-CoA epoxidase, subunit C	A>T	Non-synonymous	No	Ref	X				
	*gutB*	*ycjQ srlB*	Zinc-type alcohol dehydrogenase	G>A	Synonymous	No	Ref				X	
	unknown		Hypothetical protein	A>T	Non-sense	No	Ref				X	
	*nanK*	*yhcI*	N-acetylmannosamine kinase	Deletion TT	Frameshift	No	Ref				X	
	*mdfA*	*cmlA, cmr*	Multidrug transporter	T>C	Synonymous	No	Ref				X	
	*intE*		Prophage integrase	T>A	Non-synonymous	No	Ref				X	
	*slt*		Soluble lytic murein transglycosylase	C>T	Synonymous	No	Ref				X	
	intergenic			T>A	Intergenic	No	Ref				X	
**T6**	*yghW*		Uncharacterized protein	C>T	Non-synonymous	No	Ref				X	
	*thiC*		Hydroxymethylpyrimidine phosphate synthase	A>G	Non-synonymous	No	Ref	X			X	
**T7**	*pqiC*	*ymbA*	Polypeptide intermembrane transport lipoprotein	A>G	Non-synonymous	No	Ref					X
	*lacA*	*wbbJ*	Galactosyl O acetyl transferase	Insertion	Frameshift	No	Ref					X
**T8**	*bcr*	*bicA, bicR, suxA*	Multidrug pump efflux	C>T	Non-synonymous	Yes		Ref			X	X
	*mutS*	*ant, plm, fdv*	DNA mismatch repair protein	C>T	Non-synonymous	Yes		Ref			X	X
	*htrE*	*yehB*	Outer-membrane usher protein	G>C	Non-synonymous	Yes		Ref			X	X
	*citT*		Citrate/succinate antiporter	G>A	Synonymous	Yes		Ref			X	X
	*angR*	*irp2, mbtP*	Iron aquisition yersiniabactin synthesis enzyme	T>C	Non-synonymous	Yes		Ref	X	X	X	X
	unknown		Hypothetical protein	T>C	Synonymous	Yes		Ref		X	X	X
	*cusC*	*ibeB*, *ylcB*	Copper/silver export system outer-membrane channel protein	G>C	Non-synonymous	No		Ref	X			
**T9**	*evgS*		Hybrid sensory histidine kinase	G>A	Synonymous	No		Ref			X	
	*ypjD*		Uncharacterized protein	C>T	Synonymous	Yes		Ref		X	X	
	*murG*		N-acetylglucosaminyl transferase	C>G	Non-synonymous	Yes		Ref	X	X	X	
	*gltR*	*hdfR, gltC*	HTH-type transcriptional regulator	G>A	Non-synonymous	No		Ref	X			

Hatched cases: the persistent strain has not been detected at this follow-up point.

#### K-mer-based comparison

**Fig. 4.**
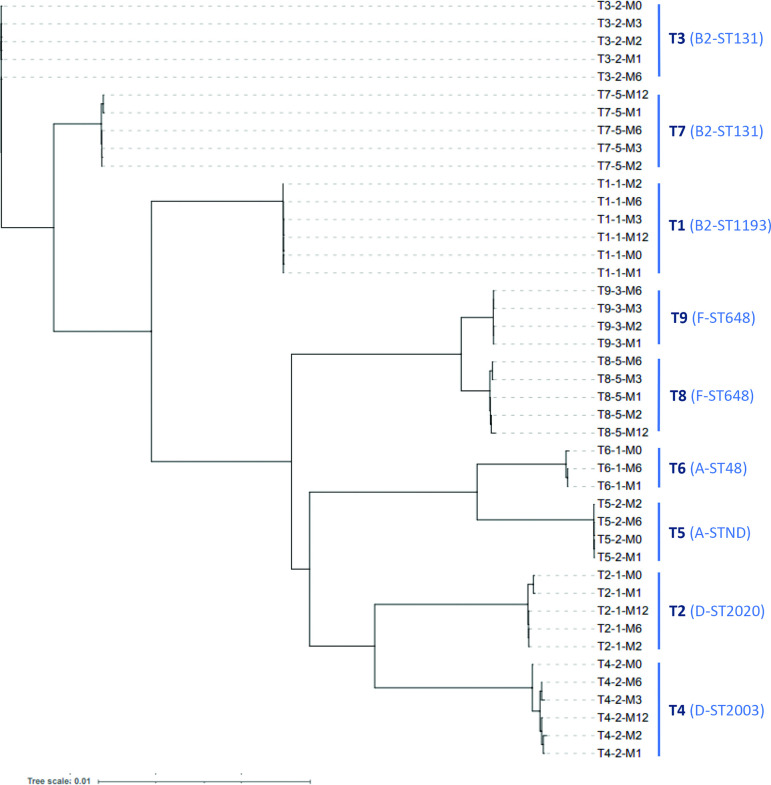
Genetic similarity between the different isolates of the nine persistent ESBL *E. coli* strains. The tree was constructed from the similarity matrix generated by the k-mer comparison approach. The tree was unrooted. Scale bar represents the distance between isolates. Phylogroup and sequence type are mentioned in parentheses for each persistent strain.

The global genome comparison based on the k-mer approach showed a very high similarity between the different isolates of the persistent strain of four travellers (T1, T3, T5 and T9) and some slight differences between the isolates of the persistent strain of five travellers (T2, T4, T6, T7 and T8) (Fig. 4). As very few SNPs have been evidenced, these differences may be explained by the loss or gain of total or partial genomic mobile elements, prophage in T2, integron or transposon (containing multiple resistance genes) in T4 and T7, and plasmid in T4, T6 and T8 (Table 3).

**Table 3.** Regions of genomic plasticity observed in the genome of the persistent ESBL *E. coli* strains during the follow-up

Traveller strains	Mobile elements	Resistance genes	Virulence genes	Size (bp)	M0	M1	M2	M3	M6	M12
**T1-1**	None	None	None							
**T2-1**	Phage	None	None	94 369	**P**	**P**				
**T3-2**	None	None	None							
**T4-2**	IncI1 plasmid	None	None	143 151		**V**	**V**	**V**	**V**	**V**
**T5-2**	Integron/transposon	*dfrA17, sul1, aadA5, mph(A), ermB*	None	9845	**P**	**P**				
	None	None	None							
**T6-1**	IncF plasmid (part)	None	None	61 078		**P**			**P**	
**T7-5**	Integron	*dfrA17, aadA5, sul1, mph(A), tet(A), strB, strA, sul2, acc(3)-IId*	None	15 452			**P**	**P**	**P**	
**T8-5**	IncL/M plasmid	None	None	53 063				**P**	**P**	
**T9-3**	None	None	None							

None, absence of variation; **P**, presence; **V**, presence with variable size between isolates; the absence of indication signifies absence of the element.

Hatched cases: the persistent strain has not been detected at this follow-up point.

### Potential ESBL plasmid transmission in individual gut microbiota

Plasmids were characterized (ESBL gene type, replicons, pMLST type) with regard to potential plasmid transmission of ESBL genes between strains isolated from the gut of each traveller. Plasmids were characterized with regard to potential plasmid transmission of ESBL genes between strains isolated from the gut of each traveller. In the 10 travellers (T2 to T11) from whom several strains were detected, neither identical ESBL enzymes (T2 to T4 and T10) nor identical plasmids (T6 to T7 and T11) nor similar location (chromosomic/plasmidic) of the ESBL gene (T9) were observed between the different strains isolated in a single traveller (Fig. 1). This suggests that no emergence of ESBL-E appears to result from gene transfer from a travel-acquired strain to a resident strain within the gut of each carrier, but does not exclude ESBL gene transfer via other genetic mobile elements, such as insertion sequences (ISs), integrons, or transposons.

## Discussion

It is now well established that travel to the tropics is associated with a high rate of ESBL-E acquisition and that carriage is usually short-lived. However, some travellers display prolonged carriage [2]. Here, we showed that long-term ESBL-E carriage following travel is associated with the acquisition of epidemic clones of *E. coli* belonging mainly to the phylogenetic groups B2/D/F and harbouring specific genetic traits (adhesins, iron capture systems and toxins) that allow them to successfully colonize their host.

There is no consensual definition of long-term carriage, which varies from several weeks to several years depending on the studies [7, 27, 28]. Because in our original study 96 % of the travellers were carriage-free 3 months following return, we considered long-term carriers to be those still carrying ESBL-E after more than 3 months. Only 11 of the 292 returning carriers met this criterion. Although multiple strains could be acquired by a single traveller, we observed that in 9 out of 11 the long-term carriage was due to colonization by a single persistent strain. Follow-up was interrupted if a new trip to a tropical region was reported, but several subjects declared travel outside this destination. Thus, some subjects may have acquired new strains in France or Europe. Even if this risk is low, given the low prevalence of ESBL-E carriage in France (5–10 %) [29, 30], this event is possible, especially as travellers represent a population at higher risk (non-French people, in contact with other people frequently travelling abroad and who may consume food products coming from high-prevalence areas).

All persistent strains were *E. coli*, the natural host of the human microbiota colonizing the microbiota at high concentrations (10^7^–10^8^) [5]. Several hypotheses might explain this prolonged carriage. One possibility is that a travel-acquired ESBL-E strain transferred its ESBL gene to a commensal *E. coli*, which was already adapted to its host. Indeed, in three travellers, the persistent strain was not isolated upon return but 1 month later. However, this hypothesis is hardly conceivable, as we found no evidence of plasmid transfer between strains isolated in a single traveller. Transfer of ESBL plasmids is known to occur between *Enterobacterales* species within the intestinal microbiota, but the frequency of this phenomenon remains unknown [31, 32]. Further, low initial concentrations could explain why the persistent strain was not initially detected at M0 in three subjects.

A second hypothesis explaining long-term carriage is that the travel-acquired ESBL *E. coli* strain subsequently evolved and adapted to the host microbiota. Investigation along this path revealed that persistent strains in the guts of travellers accumulated a maximum of eight SNPs per genome over a 12-month period. This mutation rate is consistent with commensal *E. coli* and in line with a previous study that reported up to six SNPs in clones isolated 4 or 7 months apart [33]. Analysis of the mutated genes showed a ratio of non-synonymous to synonymous mutations of 0.81 and very few convergences or fixed mutations, which is more consistent with a genetic drift rather than with an adaptation process and thus poorly supports a significant adaptation of the acquired strain to the gut of the hosts [34]. In addition, we observed genomic plasticity in some strains in which mobile genetic elements, such as integron, transposon or plasmid, may be absent or present in isolates at different follow-up points. It was not possible to determine the exact chronology of gain/loss of these elements as we studied only one isolate per sampling. Furthermore, it cannot be excluded that the observed diversity is the result of laboratory subculture and/or storage. Metapopulation analyses [35] coupled with *in vitro* fitness assays [36], should be performed in order to arrive at a better understanding of the evolutionary forces at play.

Thus, the most likely hypothesis is that the ESBL *E. coli* acquired during the trip already harboured the genetic factors required for successful adaptation to the host. We took advantage of the simultaneous carriage within the same microbiota of strains with different carriage durations, mimicking a natural competitive environment. We observed that ESBL *E. coli* belonging to extra-intestinal-associated B2/D/F phylogenetic groups have a longer duration of carriage than those belonging to the commensal groups A/B1/E. *E. coli* belonging to group B2 and, to a lesser extent, group D are known to be more pathogenic but also to colonize more efficiently [8, 27, 37]. In addition, persistent *E. coli* belonged to highly epidemic clonal complexes such as B2-STc131, B2-STc14/ST1193, d-STc69 (CGA), d-STc38 and F-STc648, all of them being frequently reported as ESBL producers [26, 38–40]. Genetic traits associated with persistent carriage were investigated in a targeted manner by comparing 42 resistance and 149 virulence genes, and with a non-targeted approach encompassing the entire complement of the genes identified after sequencing. Both methods confirmed that persistent strains harboured more virulence factors in their genome than transient strains. While 50–60 % of the commensal *E. coli* [8, 41] carried *fyuA/irp* genes, all persistent ESBL *E. coli* had these genes irrespective of their phylogenetic group. *fyuA/irp* encode the yersiniabactin siderophore, an iron capture system belonging to the HPI, which has already been associated with longer duration of carriage, although not at such a high frequency [8]. Genes coding for molecules involved in the adhesion process were found much more often in persistent strains. These included adhesins belonging to the Afa/Dr family (*daaF* and *afaA/nfaE)* [42], the self-recognizing adhesin, antigen 43 involved in cell aggregation (*flu)* [43], the P fimbriae involved in adherence in pyelonephritis (*pap* operon) [44] and the common *E. coli* pilus (*ecpA*) [44]. This pattern of prevalence also held for genes coding for the type VI secretion system (*vasA/vasB*), which could be involved in cell interaction and biofilm formation [45], and toxins such as *sat* encoding the secreted autotransporter toxin, a vacuolating cytotoxin [46] or *senB* encoding the enterotoxin TieB. As already described, the *senB* gene was associated with the *crj* operon encoding a colicin [47]. Extra-intestinal virulence genes coding for adhesins, iron capture systems and toxins have previously been correlated with successful gut colonization in humans [6]. Our results confirm that virulence factors are not only involved in strain pathogenicity, but also in commensalism [48, 49]. An important result is that the ‘virulence factors’ associated with persistence were found in both the widely described B2 strains as well as in the D, F and A persistent strains. These so-called virulence factors improve adaptability and competitiveness and may promote longer intestinal colonization [6, 9, 50]. It is a matter of concern when these factors are present in ESBL-E as, by facilitating longer carriage, they also contribute to a higher risk of transmission and therefore dissemination of ESBL-E. These results concerning genetic factors specific to the bacteria must be understood in a global context, integrating the role and the complex interactions between the environment, the host and its microbiota, as they are inextricably linked [5].

Our study has several limitations. First, the modest size of our study, including the number of travellers with long-term carriage, as well as the number of persistent and transient strains, limits the statistical power of the analyses. Nevertheless, we have been able to correlate our findings with similar observations in the literature. Second, we took only one morphologically different colony per plate, which is a limitation in investigating the diversity of ESBL-E carriage. This would require between 5 and 15 colonies per plate to detect subdominant clones, or a metagenomic approach [35]. Third, even though we used an enrichment step, which increases the sensitivity of the culture, strains colonizing the gut microbiota at low concentrations may have been missed. Moreover, since no molecular methods were performed directly in the stool samples, we could not exclude the possibility that the ESBL-E genes could be carried and transmitted from viable but nonculturable (VBNC) bacteria. Fourth, as no samples were taken during travel, we could not determine the exact timing of the acquisition and could not exclude the possibility that rapid strain adaptations (plasmid or mobile element transfer, mutations) may have occurred during the trip. Finally, no household members or pets have been investigated for ESBL-E colonization. Even if the risk seems low, we cannot exclude the possibility that the persistence of carriage may be due to iterative acquisition of the same strain circulating within the household. Thus, the association between the acquisition of preadapted multi-resistant *E. coli* clones and long-term carriage after travel abroad should be confirmed in further and larger studies.

In conclusion, long-term colonization by ESBL-E following travel appears to be associated with the acquisition of *E. coli* strains belonging to epidemic clones and harbouring specific traits, including extra-intestinal virulence genes, allowing good adaptation to the intestinal microbiota.

## Supplementary Data

Supplementary material 1

Supplementary material 2
