# Supplementary material for: Dynamics of extended-spectrum beta-lactamase-producing Enterobacterales colonization in long-term carriers following travel abroad
Source: Microb Genom. 2021 Jul 19;7(7):000576. doi: 10.1099/mgen.0.000576 (PMC8477403; doi:10.1099/mgen.0.000576)
Supplement: Supplementary material 1 [file mgen-7-0576-s001.pdf]

Table S1. Description of the 11 travellers and data collected before travel, after travel and during the follow-up.

	T1	T2	T3	T4	T5	T6	T7	T8	T9	T10	T11
Age	31	57	27	53	22	52	19	40	29	31	21
Sex	F	F	F	F	M	F	F	F	F	M	F
Area of travel	Asia	Latin America	Latin America	Asie	Asia	Asia	Asia	Asia	Latin America	Asia	Asia
Country of travel	Thailand	Ecuador	Peru	Vietnam	India	India	Vietnam	India	Brazil	Vietnam	India
Duration of the trip	16	22	14	15	89	21	18	16	22	14	41
Previous travel to the tropics the year before	No	No	No	No	No	No	No	No	No	No	No
Type of travel	Backpacking	Family / Backpacking	Organized tour	Organized tour	Backpacking	Backpacking	Organized tour	Backpacking	Organized tour	Backpacking	Family / Backpacking
Vegetarian diet	No	No	No	No	No	No	No	No	No	No	No
Antimalarial	Yes	Yes	Yes	No	Yes	Yes	Yes	Yes	No	No	Yes
**During travel**											
Diarrhea	No	No	No	No	No	No	Yes	No	No	No	Yes
Antibiotic consumption	No	Yes	Yes	No	No	No	No	No	No	No	No
Type of antibiotic		Cloxacillin									
Contact with healthcare structure	No	Yes	No	No	No	No	No	No	No	No	No
**M0-M1 Follow-up**											
Travels	No	No	No	No	Yes	No	No	No	No	No	No
Country					NS						
Antibiotic consumption	No	No	No	No	No	Yes	Yes	No	No	No	No
Type of antibiotic						Amoxicillin	Amoxicillin				
Infectious disease						Emesis	Rhinopharyngitis				
Hospitalization	No	No	No	No	No	No	No	No	No	No	No
**M1-M2 Follow-up**											
Travels	No	No	No	No	No	No	No	No	Yes	NA	No
Country									Spain		
Antibiotic consumption	No	No	No	No	No	Yes	No	No	Yes	No	No
Type of antibiotic						Co-amoxiclav			Ciprofloxacin		
Infectious disease						Sinusitis			Urinary tract infection		
Hospitalization	No	No	No	No	NA	No	No	No	No	NA	No
**M2-M3 Follow-up**											
Travels	No	No	No	No	No	No	No	No	No	No	No
Antibiotic consumption	No	No	No	No	No	No	No	No	No	No	No
Hospitalization	No	No	No	No	No	No	No	No	No	No	No
**M3-M6 Follow-up**											
Travels	No	No	Yes	NA	Yes	No	No	No	No	No	No
Country			NS		NS						
Antibiotic consumption	No	No	No	NA	No	No	No	No	Yes	No	No
Type of antibiotic									Norfloxacin		
Infectious disease									Urinary tract infection		
Hospitalization	No	No	No	NA	No	No	No	No	No	No	No
**M6-M12 Follow-up**											
Travels	No	No	No	No	NA	Yes	Yes	No	Yes	NA	Yes
Country						NS	NS		NS		NS
Antibiotic consumption	Yes	No	No	No	NA	No	No	No	No	NA	Yes
Type of antibiotic	Cefpodoxime										Amoxicillin
Infectious disease	NS										Angina
Hospitalization	No	No	No	No	NA	No	No	No	No	NA	No

** NS : not specified, NA: not available*

Table S2. Description of all isolates

	Present strain ID	Original strain ID	Carriage duration	Transient/persistent status	Phylogenetic group	ST Warwick	STc Warwick	ST Pasteur	Serotype	fimH allele	ESBL enzyme	Accession number
T1	T1-1	C4457 S2 ECR	12	Persistent	B2	1193	14	53	O75:H5	fimH64	CTX-M-27	ERS5275910
	T1-1	C4457 M1 EC	12	Persistent	B2	1193	14	53	O75:H5	fimH64	CTX-M-27	ERS5275911
	T1-1	C4457 M2 EC	12	Persistent	B2	1193	14	53	O75:H5	fimH64	CTX-M-27	ERS5275912
	T1-1	C4457 M3 EC	12	Persistent	B2	1193	14	53	O75:H5	fimH64	CTX-M-27	ERS5275913
	T1-1	C4457 M6 EC	12	Persistent	B2	1193	14	53	O75:H5	fimH64	CTX-M-27	ERS5275914
	T1-1	C4457 M12 EC	12	Persistent	B2	1193	14	53	O75:H5	fimH64	CTX-M-27	ERS5275915
T2	T2-1	C4254 S2 EC2	12	Persistent	D	2020	69	3	O11:H4	fimH27	CTX-M-55	ERS5275916
	T2-1	C4254 M1 EC	12	Persistent	D	2020	69	3	O11:H4	fimH27	CTX-M-55	ERS5275917
	T2-1	C4254 M2 EC	12	Persistent	D	2020	69	3	O11:H4	fimH27	CTX-M-55	ERS5275918
	T2-2	C4254 M3 EC1	ND		A	2325	467	645	O56:H25	fimH40	CTX-M-14	ERS5275919
	T2-1	C4254 M6 EC	12	Persistent	D	2020	69	3	O11:H4	fimH27	CTX-M-55	ERS5275920
	T2-1	C4254 M12 EC	12	Persistent	D	2020	69	3	O11:H4	fimH27	CTX-M-55	ERS5275921
T3	T3-1	C5016 S2 EC1	<1	Transient	A	638	10	378	O101/162:H33	fimHneg	CTX-M-3	ERS5275922
	T3-2	C5016 S2 EC2	6	Persistent	B2	131	131	43	O25:H4	fimH30	CTX-M-15	ERS5275923
	T3-2	C5016 M1 EC	6	Persistent	B2	131	131	43	O25:H4	fimH30	CTX-M-15	ERS5275924
	T3-2	C5016 M2 EC	6	Persistent	B2	131	131	43	O25:H4	fimH30	CTX-M-15	ERS5275925
	T3-2	C5016 M3 EC	6	Persistent	B2	131	131	43	O25:H4	fimH30	CTX-M-15	ERS5275926
	T3-2	C5016 M6 EC	6	Persistent	B2	131	131	43	O25:H4	fimH30	CTX-M-15	ERS5275927
T4	T4-1	C4059 S2 EC2	1		F	648	648	ND	O1:H6	fimHneg	CTX-M-27	ERS5275928
	T4-2	C4059 S2 EC1	12	Persistent	D	2003	38	8	O1:H15	fimH65	CTX-M-55	ERS5275929
	T4-1	C4059 M1 EC2	1		F	648	648	ND	O1:H6	fimHneg	CTX-M-27	ERS5275930
	T4-2	C4059 M1 EC1	12	Persistent	D	2003	38	8	O1:H15	fimH65	CTX-M-55	ERS5275931
	T4-2	C4059 M2 EC	12	Persistent	D	2003	38	8	O1:H15	fimH65	CTX-M-55	ERS5275932
	T4-2	C4059 M3 EC	12	Persistent	D	2003	38	8	O1:H15	fimH65	CTX-M-55	ERS5275933
	T4-2	C4059 M6 EC	12	Persistent	D	2003	38	8	O1:H15	fimH65	CTX-M-55	ERS5275934
	T4-2	C4059 M12 EC	12	Persistent	D	2003	38	8	O1:H15	fimH65	CTX-M-55	ERS5275935
T5	T5-1	C4379 S2 EC2	2		A	6143	10	638	Oneg:H10	fimHneg	CTX-M-15	ERS5275936
	T5-2	C4379 S2 EC1	6	Persistent	A	ND	10	999	O92:H5	fimH559	CTX-M-15	ERS5275937
	T5-1	C4379 M1 EC2	2		A	6143	10	638	Oneg:H10	fimHneg	CTX-M-15	ERS5275938
	T5-2	C4379 M1 EC1	6	Persistent	A	ND	10	999	O92:H5	fimH559	CTX-M-15	ERS5275939
	T5-1	C4379 M2 EC2	2		A	6143	10	638	Oneg:H10	fimHneg	CTX-M-15	ERS5275940
	T5-2	C4379 M2 EC1	6	Persistent	A	ND	10	999	O92:H5	fimH559	CTX-M-15	ERS5275941
	T5-3	C4379 M3 EC	ND		D	38	38	535	O7:H18	fimH65	CTX-M-1	ERS5275942
	T5-2	C4379 M6 EC	6	Persistent	A	ND	10	999	O92:H5	fimH559	CTX-M-15	ERS5275943
T6	T6-1	C4405 S2 EC	6	Persistent	A	48	10	132	Oneg:H30	fimHneg	CTX-M-15	ERS5275944
	T6-1	C4405 M1 EC2	6	Persistent	A	48	10	132	Oneg:H30	fimHneg	CTX-M-15	ERS5275945
	T6-2	C4405 M1 EC1	2		B2	131	131	43	O25:H4	fimH30	CTX-M-15	ERS5275946
	T6-2	C4405 M2 EC2	2		B2	131	131	43	O25:H4	fimH30	CTX-M-15	ERS5275947
	T6-3	C4405 M2 EC1	1		A	656	10	ND	O89:H45	fimH54	CTX-M-15	ERS5275948
	T6-2	C4405 M3 EC1	2		B2	131	131	43	O25:H4	fimH30	CTX-M-15	ERS5275949
	T6-3	C4405 M3 EC2	1		A	656	10	ND	O89:H45	fimH54	CTX-M-15	ERS5275950
	T6-1	C4405 M6 EC	6	Persistent	A	48	10	132	Oneg:H30	fimHneg	CTX-M-15	ERS5275951

T7	T7-1	C4255 S2 EC1	<1	Transient	A	189	165	809	O112ab:H26	fimH54	CTX-M-55	ERS5275952
	T7-2	C4255 S2 EC2	<1	Transient	F	1722	1722	ND	O1:H25	fimH153	CTX-M-27	ERS5275953
	T7-3	C4255 S2 EC3	<1	Transient	B1	155	155	21	O179:H51	fimH32	CTX-M-55	ERS5275954
	T7-4	C4255 S2 EC4	<1	Transient	B2	131	131	506	O25:H4	fimH41	CTX-M-27	ERS5275955
	T7-5	C4255 M1 EC1	11	Persistent	B2	131	131	506	O16:H5	fimH41	CTX-M-27	ERS5275956
	T7-5	C4255 M1 EC2	11	Persistent	B2	131	131	506	O16:H5	fimH41	CTX-M-27	ERS5275957
	T7-5	C4255 M2 EC	11	Persistent	B2	131	131	506	O16:H5	fimH41	CTX-M-27	ERS5275958
	T7-5	C4255 M3 EC	11	Persistent	B2	131	131	506	O16:H5	fimH41	CTX-M-27	ERS5275959
	T7-5	C4255 M6 EC	11	Persistent	B2	131	131	506	O16:H5	fimH41	CTX-M-27	ERS5275960
	T7-5	C4255 M12 EC1	11	Persistent	B2	131	131	506	O16:H5	fimH41	CTX-M-27	ERS5275961
T8	T8-1	C1152 S2 EC2	<1	Transient	A	656	10	ND	Oneg:H4	fimHneg	CTX-M-15	ERS5275962
	T8-2	C1152 S2 EC3	<1	Transient	A	2325	467	ND	O51:H10	fimH54	CTX-M-15	ERS5275963
	T8-2	C1152 S2 EC4	<1	Transient	A	2325	467	ND	O51:H10	fimH54	CTX-M-15	ERS5275964
	T8-3	C1152 S2 EC5	<1	Transient	A	46	46	398	O9:H4	fimH34	CTX-M-15	ERS5275965
	T8-4	C1152 S2 EC1	1		A	44	10	2	O89:H4	fimH54	CTX-M-15	ERS5275966
	T8-4	C1152 M1 EC2	1		A	44	10	2	O89:H4	fimH54	CTX-M-15	ERS5275967
	T8-5	C1152 M1 EC1	11	Persistent	F	648	648	ND	O1:H6	fimH27	CTX-M-15	ERS5275968
	T8-5	C1152 M2 EC	11	Persistent	F	648	648	ND	O1:H6	fimH27	CTX-M-15	ERS5275969
	T8-5	C1152 M3 EC	11	Persistent	F	648	648	ND	O1:H6	fimH27	CTX-M-15	ERS5275970
	T8-5	C1152 M6 EC	11	Persistent	F	648	648	ND	O1:H6	fimH27	CTX-M-15	ERS5275971
	T8-5	C1152 M12 EC1	11	Persistent	F	648	648	ND	O1:H6	fimH27	CTX-M-15	ERS5275972
	T8-5	C1152 M12 EC2	11	Persistent	F	648	648	ND	O1:H6	fimH27	CTX-M-15	ERS5275973
T9	T9-1	C4500 S2 EC1	<1	Transient	F	648	648	ND	O1:H6	fimH5	CTX-M-15	ERS5275974
	T9-2	C4500 S2 EC2	2		D	405	405	477	O102:H6	fimH27	CTX-M-27	ERS5275975
	T9-3	C4500 M1 EC	5	Persistent	F	648	648	ND	O102:H6	fimH5	CTX-M-15	ERS5275976
	T9-3	C4500 M2 EC1	5	Persistent	F	648	648	ND	O102:H6	fimH5	CTX-M-15	ERS5275977
	T9-2	C4500 M2 EC2	2		D	405	405	477	O102:H6	fimH27	CTX-M-27	ERS5275978
	T9-3	C4500 M3 EC	5	Persistent	F	648	648	ND	O102:H6	fimH5	CTX-M-15	ERS5275979
	T9-3	C4500 M6 EC	5	Persistent	F	648	648	ND	O102:H6	fimH5	CTX-M-15	ERS5275980
	T9-4	C4500 M12 EC	ND		A	167	10	2	O89:H21	fimH54	CTX-M-15	ERS5275981
T10	T10-1	C4322 S2 EC	<1	Transient	A	165	165	865	Oneg:H52	fimH398	CTX-M-15	ERS5275982
	T10-2	C4322 M1 EC	2		D	38	38	8	O86:H18	fimHneg	CTX-M-27	ERS5275983
	T10-2	C4322 M2 EC	2		D	38	38	8	O86:H18	fimHneg	CTX-M-27	ERS5275984
	T10-2	C4322 M3 EC	2		D	38	38	8	O86:H18	fimHneg	CTX-M-27	ERS5275985
	T10-3	C4322 M6 EC	ND		B1	448	448	56	O8:H8	fimH35	CTX-M-55	ERS5275986
T11	T11-1	C4170 S2 EC2	<1	Transient	E	2064	2064	ND	O52:H45	fimH54	CTX-M-15	ERS5275987
	T11-2	C4170 S2 EC1	1		A	ND	10	998	Oneg:H30	fimH54	SHV-12	ERS5275988
	T11-2	C4170 M1 EC1	1		A	ND	10	998	Oneg:H30	fimH54	SHV-12	ERS5275989
	T11-3	C4170 M1 EC3	ND		A	43	10	ND	O95:H10	fimHneg	CTX-M-15	ERS5275990
	T11-4	C4170 M1 EC2	2		A	227	10	638	O92:H10	fimHneg	CTX-M-15	ERS5275991
	T11-4	C4170 M2 EC	2		A	227	10	638	O92:H10	fimHneg	CTX-M-15	ERS5275992
	T11-4	C4170 M3 EC	2		A	227	10	638	O92:H10	fimHneg	CTX-M-15	ERS5275993
	T11-5	C4170 M6 EC	ND		A	5420	168	83	O88:H28	fimH27	SHV-12	ERS5275994
